# Differential Associations of Anticipatory and Consummatory Anhedonia With Depression and Social Anxiety Symptoms: A Network Analysis of University Students

**DOI:** 10.1155/da/5674096

**Published:** 2025-10-30

**Authors:** Sakshi Rajesh, Sverre Urnes Johnson, Asle Hoffart, Eleanor Leigh, Omid V. Ebrahimi

**Affiliations:** ^1^Department of Psychiatry, University of Oxford, Oxford, UK; ^2^Department of Psychology, University of Oslo, Oslo, Norway; ^3^Modum Bad Psychiatric Center and Research Institute, Vikersund, Norway; ^4^Department of Experimental Psychology, University of Oxford, Oxford, UK

**Keywords:** anhedonia, depression, network analysis, social anxiety, young people

## Abstract

**Background:**

Depression and social anxiety are frequently co-occurring conditions that significantly impact young people. Anhedonia may be important to consider in early interventions for these conditions, but the roles of specific dimensions of anhedonia—anticipatory and consummatory—are not well understood. This study explored the symptom connectivity of depression and social anxiety in university students, focusing on how the two dimensions of anhedonia relate to symptoms of both conditions.

**Methods:**

We conducted a cross-sectional network analysis of data from 672 university students (19–24 years). A Gaussian graphical model was used to investigate the relationship between anticipatory and consummatory anhedonia and symptoms of depression and social anxiety.

**Results:**

Anticipatory anhedonia was distinctively connected with specific depression nodes (*low mood* and *suicidal ideation*) and social anxiety nodes (*avoiding being the centre of attention* and *less fear of embarrassment*). Consummatory anhedonia was related to a wider range of depression nodes (*worthlessness/guilt, suicidal ideation*, *concentration problems* and *sleep problems*) and all social anxiety nodes. Both dimensions of anhedonia demonstrated strong bridge expected influence (EI), alongside *worthlessness/guilt* and *avoiding being the centre of attention*, highlighting their relevance to both social anxiety and depression nodes.

**Conclusions:**

The findings refine our understanding of the psychopathology of depressive and social anxiety symptoms in young people and exemplify the importance of distinguishing the dimensions of anhedonia. Given its transdiagnostic associations, anhedonia may be important to account for in early interventions for depression and social anxiety. Future research should incorporate clinical samples and longitudinal data.

## 1. Introduction

Depression and social anxiety are commonly co-occurring conditions, with co-occurrence rates ranging from 35% to 70% [1]. Together, they have been associated with compounding negative consequences in a range of areas, including psychosocial functioning, occupational functioning, suicidal ideation and substance abuse [2, 3]. Depression and social anxiety both emerge early in life, with peak ages of onset at 19.5 and 14.5 years, respectively [4]. Given their early onset, both conditions are highly prevalent in university students [5, 6], who are at an increased risk of experiencing mental health difficulties due to transitional challenges, academic demands and social pressures [7, 8]. University students are also less likely to respond to routine psychological treatment for depression and anxiety (including social anxiety), compared to same-age employed peers [9]. Together, these findings underscore the importance of early interventions for depression and social anxiety.

To inform early interventions, we may identify symptoms that are connected to both depression and social anxiety, and one candidate that merits exploration is anhedonia. Anhedonia refers to an enduring 'markedly diminished interest or pleasure in all, or almost all, activities' [10]. It has been receiving increasing consideration in a range of mental health conditions and is often considered a transdiagnostic symptom (i.e., holds relevance to several different mental health conditions, cutting across diagnostic boundaries) [11]. It is a hallmark symptom of depression [10] and has been observed to a significant magnitude in social anxiety [12, 13]. It is associated with outcomes such as poorer psychosocial functioning [14] and increased suicidal ideation [15], with these being prominent in university students [16]. Considering its relevance and consequences, anhedonia may be a promising early interventional candidate for depression and social anxiety, and it is worth developing our understanding of how anhedonia relates to both conditions. However, we do not yet have a precise understanding of this, as studies have tended to conceptualise anhedonia monolithically. Anhedonia is a multidimensional symptom, encompassing a range of reward-related components [17]. We can distinguish between anticipatory anhedonia—i.e., a persistent reduction in pleasure one expects to experience from future rewards—and consummatory anhedonia—i.e., a persistent reduction in momentary pleasure obtained upon receiving rewards [18]. This distinction may capture the phenomenology of anhedonia more accurately, given neuroimaging [19, 20] and behavioural [21, 22] evidence suggesting that these constructs are dissociable, such that anticipatory anhedonia is observed, but consummatory anhedonia is less so.

Network approaches serve as a useful way to improve our understanding of the relationships between these dimensions of anhedonia and symptoms of depression and social anxiety. The network theory of mental disorders conceptualises mental disorders as interconnected networks of symptoms [23–27], comprising bridge symptoms that facilitate the spread of symptom activation across conditions [28]. Crucially, previous network studies have identified anhedonia as a ‘bridge symptom' between these conditions [29, 30]. This suggests that anhedonia may act as a shared vulnerability factor between depression and social anxiety or serve as a pathway from one condition to the other, supporting its transdiagnostic conceptualisation. For example, social anxiety may result in less pleasure from social situations, which may result in a depressed mood. Likewise, low mood arising from depression may hinder individuals from enjoying social interactions, which may exacerbate self-consciousness as observed in social anxiety. Examining these constructs on a symptom level could provide insights into the psychopathological expression of these conditions to help guide early interventional strategies. For example, anticipatory anhedonia is well addressed through techniques like pleasant event scheduling [31, 32]; conversely, consummatory anhedonia may require strategies such as present-moment imaginal recounting [33, 34].

Building on this, the present study employed a network approach to elucidate connectivity across depressive and social anxiety symptoms in university students, with a particular focus on examining how anticipatory and consummatory anhedonia link to symptoms of both conditions.

## 2. Materials and Methods

The present study was pre-registered, and its protocol can be found on Open Science Framework (OSF; https://osf.io/c97g5).

### 2.1. Sample

The CIPA Study [35] is a nationally representative longitudinal investigation of mental health in the general Norwegian population. We used data from CIPA Students, a sub-study of CIPA focused on university students. CIPA students is a new study, and its data have not been analysed previously. We analysed data measured at the third time point in the longitudinal CIPA students study, as this was the wave wherein the Temporal Experience of Pleasure Scale (TEPS) was included. We included participants less than or equal to the age of 24^1^, in alignment with contemporary definitions of young people [36].

The sample comprised 672 university students (69.94% female, 29.17% male, 0.60% transgender and 0.30% intersex), aged 19–24 years (*M* = 22.19, *SD* = 1.43). Descriptive statistics for the relevant questionnaires are presented in Table 1. In the sample, 28.87% and 18.90% scored above the clinical cutoffs for depression (with a score of ≥10 out of 27) [37] and social anxiety (with a score of ≥6 out of 12) [38], respectively.

### 2.2. Procedure

The CIPA study received ethical approval from the Regional Committee for Medical and Health Research Ethics in Norway (reference: 522020). Data collection for CIPA students involved inviting a random sample of students from university enrolment lists across universities in Norway to participate in an online survey assessing mental health via email. All participants provided informed consent to participate in the study. Data collection for the third time point of CIPA students was completed in 2024.

### 2.3. Measures

#### 2.3.1. Depressive Symptoms

Depression symptoms were measured using the Patient Health Questionnaire (PHQ-9) [37]. This is a self-report instrument measuring how often respondents are bothered by each symptom in the past 2 weeks, with nine items rated on a 4-point Likert Scale (0: not at all—3: nearly every day). Each item was included individually as a node in the network, except for item one. As indicated in the pre-registration, item one was intentionally excluded from the network to avoid topological overlap [39], as it measures anhedonia, which was assessed using a separate, more detailed scale (TEPS), outlined subsequently. It is important to avoid topological overlap, as edge weight estimates can conflate conceptual overlap with a statistical association [39]. Hence, the following 8 symptoms from the PHQ-9 were examined: *low mood, sleep problems, low energy, appetite changes, worthlessness/guilt, concentration difficulties, psychomotor agitation/slowing* and *suicidal ideation*. The internal consistency of this scale was high (Cronbach's *α* = 0.88).

#### 2.3.2. Social Anxiety Symptoms

Social anxiety symptoms were measured using the Mini Social Phobia Inventory (Mini-SPIN) [38]. The Mini-SPIN captures the core diagnostic features of social anxiety, i.e., fear of negative evaluation and related avoidance. This is a self-report scale measuring feelings in the past week, with three items rated on a 5-point Likert Scale (0: not at all—4: extremely). Each item was included individually in the network: *avoiding embarrassment, avoiding being the centre of attention* and *fear of embarrassment*. The internal consistency of this scale was high (Cronbach's *α* = 0.85).

#### 2.3.3. Anhedonia

Anhedonia was measured using the TEPS [40]. This is a self-report scale measuring pleasure experiences in the past week, with 18 items rated on a 6-point Likert Scale (1: very false for me—6: very true for me). It comprises subscales measuring pleasure across two dimensions—anticipatory and consummatory. In contrast to the item-level approach for depressive and social anxiety symptoms, sum scores on items corresponding to these dimensions were reversed and included in the model as *anticipatory anhedonia* and *consummatory anhedonia* to investigate the associations of these dimensions of anhedonia with depressive and social anxiety symptomatology, in accordance with the pre-registered aims of this study. The internal consistencies of the subscales were high (anticipatory anhedonia: Cronbach's *α* = 0.84; consummatory anhedonia: Cronbach's *α* = 0.82).

### 2.4. Data Analysis

#### 2.4.1. Data Preparing

All analyses were performed in R (version 4.4.2). All code necessary to reproduce our analyses is provided on OSF (https://osf.io/54xyk). There were no missing data present.

#### 2.4.2. Network Estimation

A Graphical Gaussian Model (GGM) [41] was estimated, which provides the unique relationship between variables, controlling for the effects of all other variables in the network. These estimates consist of partial correlations. The GGM included 13 nodes: eight depression nodes (from the PHQ-9; all excluding anhedonia), three social anxiety nodes (from the Mini-SPIN) and two anhedonia nodes (from the TEPS).

We selected the best model using the EBICglasso2 model selection algorithm, part of the bootnet package [42]. This method applies the 'least absolute shrinkage and selection operator' (LASSO) [43], a regularisation method that adds a penalty for each variable and therefore shrinks small edge weights to zero, removing them from the model. This retains the most important relationships in the network and reduces the risk of a type I error. The method uses a tuning parameter that is varied to return 100 network models, and the Extended Bayesian Information Criterion (EBIC) [44] is used to select the best-fitting model. To control the extent of regularisation, a default hypertuning parameter of *γ* = 0.5 was used [45]. We chose the EBICglasso algorithm as per published guidelines [45], as its sensitivity to detecting weaker edges makes it most appropriate to use with sample sizes such as that of the present study. We specified the algorithm to calculate Spearman's rank-order correlation coefficients, which is the recommended approach for estimating networks with ordinal data, as it offers more stable estimates than alternative approaches [46–48].

The network was visualised using the qgraph package [49]. The layout of nodes was determined manually to ensure the ease of interpretation of nodes linking to anhedonia, which was the focus of this study.

#### 2.4.3. Centrality Indices

##### 2.4.3.1. Expected Influence

Expected influence (EI) [50] was calculated and plotted using the centralityPlot function of the qgraph package. EI evaluates how well a node is overall connected to other nodes in the network, and thus conveys information about how important a node is within a network; the higher the value, the more strongly connected the node is in the network.

##### 2.4.3.2. Bridge Expected Influence

One-step bridge EI (Bridge EI) [51] was calculated and plotted using the bridge function of the networktools package. Bridge EI evaluates the extent to which a node connects to nodes from other communities (e.g., symptom clusters) than its own; the higher the value, the greater the extent to which the node connects to other communities. We specified communities manually related to our research question, as all nodes were derived from validated questionnaires. Hence, three communities were defined: depression nodes, social anxiety nodes and anhedonia nodes.

#### 2.4.4. Network Accuracy and Stability

##### 2.4.4.1. Edge Weight Accuracy

We assessed the accuracy of estimated edge weights through non-parametric bootstrapping, using the bootnet package [42]. This method resamples from the original dataset 1000 datasets of the same size with replacement and computes edge weight statistics in each resampled dataset to draw bootstrapped confidence intervals for each edge parameter.

##### 2.4.4.2. Stability of Centrality Indices

We assessed the stability of centrality indices using case-drop bootstrapping, using the bootnet package [42]. This method calculates centrality indices in subsets of the original dataset, dropping 10% at a time, and then correlates all calculated centrality indices to produce a correlation stability coefficient (CS coefficient). The CS coefficient provides an estimate of the maximum number of cases that can be dropped from the data to retain, with 95% probability, a correlation of at least 0.70 with the original centrality indices. The CS coefficient should be a value of at least 0.50 [42].

The results are reported following the reporting standards for network analyses [39].

## 3. Results

### 3.1. Network Estimation

**Figure 1:**
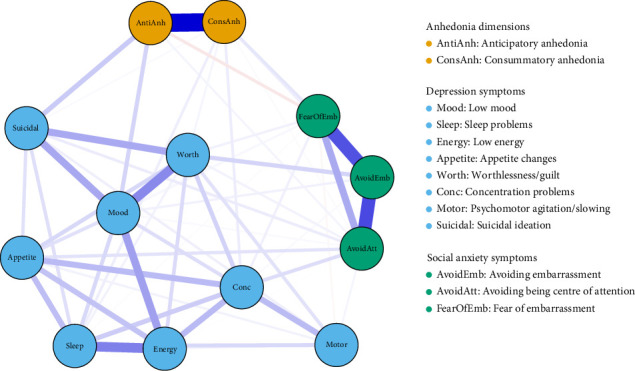
Network of dimensions of anhedonia, depression symptoms and social anxiety symptoms. Note: blue edges represent positive associations, while red edges represent negative associations. The thicker the edge, the stronger the association.

Figure 1 depicts the network model. Edges represent partial correlation coefficients between nodes, with blue edges denoting a positive and red edges denoting a negative relationship. The thickness of the edges indicates the strength of associations, with thicker edges conveying stronger relationships. Nodes clustered together by symptom group. Descriptive statistics for each node in the network and the computed edge weight matrices are available in the Supporting Information (Tables S1, S2).

*Anticipatory anhedonia* and *consummatory anhedonia* showed differential associations with depression and social anxiety nodes. With depression nodes, *anticipatory anhedonia* was strongly connected with *low mood* and *suicidal ideation*. *Consummatory anhedonia* was strongly associated with *sleep problems* and *concentration problems*, and less strongly with *worthlessness/guilt* and *suicidal ideation*. For social anxiety nodes, *anticipatory anhedonia* was positively connected with *avoiding being the centre of attention* and negatively associated with fear of embarrassment. *Consummatory anhedonia* was positively connected with all three social anxiety nodes, and more strongly with *avoiding embarrassment* and *fear of embarrassment*, than with *avoiding being the centre of attention*. Individual network visualisations for anticipatory anhedonia and consummatory anhedonia are available in the Supporting Information (Figures S4, S5).

There were additional associations across depression and social anxiety clusters, as well as notable within-cluster associations. The strongest across-cluster association was observed between *worthlessness/guilt* and *avoiding embarrassment*, followed by *concentration problems* and *avoiding being the centre of attention*, and *appetite changes* and *avoiding being the centre of attention*. Within clusters, anticipatory anhedonia and consummatory anhedonia were strongly positively associated with one another, as were all social anxiety nodes. Within depression nodes, *worthlessness/guilt, low mood* and *suicidal ideation* were strongly interrelated, as were *low energy, sleep problems, concentration problems* and *appetite changes*. *Low mood* was also associated with *low energy*, and *concentration problems* with *psychomotor agitation/slowing*. There were numerous weaker but significant associations, all of which are visualised in the network.

### 3.2. Centrality Indices

**Figure 2:**
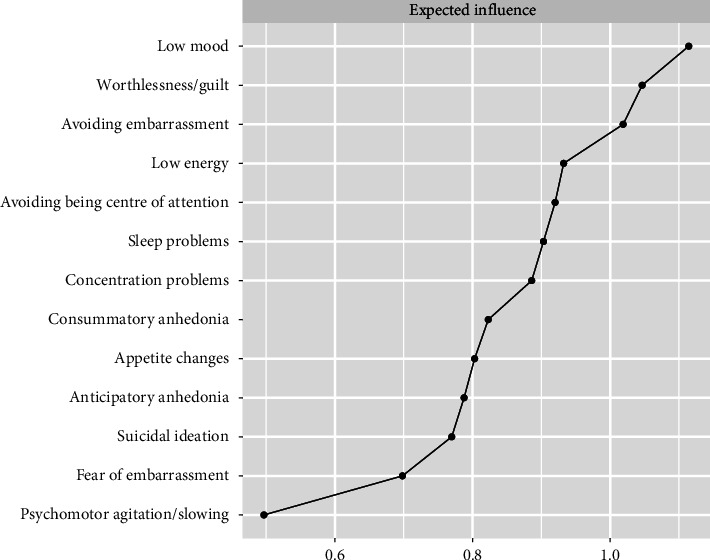
Expected influence estimates for dimensions of anhedonia, depression nodes and social anxiety nodes.

Figure 2 displays the EI of each node in the network. The nodes with the strongest EI were *low mood, worthlessness/guilt* and *avoiding embarrassment*. The nodes with the weakest EI were *psychomotor agitation/slowing, fear of embarrassment* and *suicidal ideation*.

**Figure 3:**
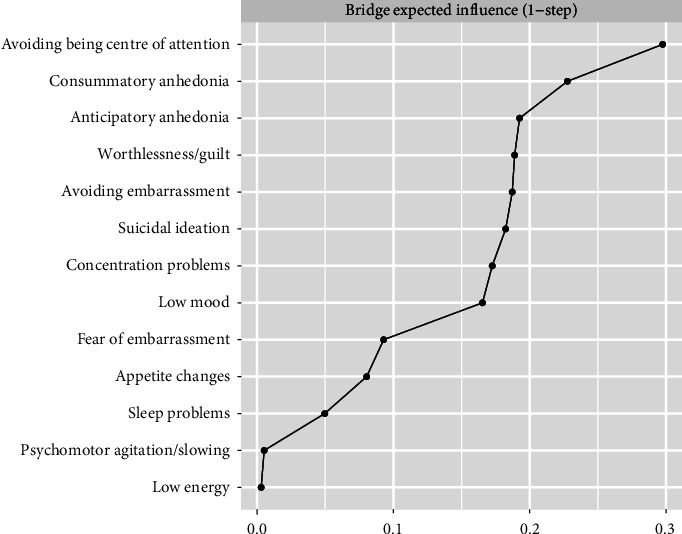
Bridge expected influence estimates for dimensions of anhedonia, depression nodes and social anxiety nodes.

Figure 3 shows the bridge EI of each node in the network. The nodes with the strongest bridge EI were avoiding being the centre of attention, consummatory anhedonia, anticipatory anhedonia and worthlessness/guilt.

### 3.3. Network Accuracy and Stability

Non-parametric bootstrapping and case-drop bootstrapping results are available in the Supporting Information (Figures S6–S8). These analyses indicated a high accuracy of edge weights and stability of centrality indices, attesting to the robustness of the estimated network. The CS coefficients were 0.75 for EI estimates and 0.59 for bridge EI estimates, both above the recommended stability threshold of 0.50.

## 4. Discussion

This study aimed to examine the connectivity across depressive and social anxiety symptoms in university students, with particular focus on whether anticipatory and consummatory anhedonia showed differential associations within and across symptom clusters. The network analysis indicated that at the symptom level, both dimensions of anhedonia were associated with symptoms of depression and social anxiety, with differences being pronounced in the pattern of associations.

Differences in the pattern of associations may be explained by processes of reward anticipation and consumption. Reward anticipation is inherently a top-down process that requires prior knowledge to predict the incidence and valence of rewards arising from future experiences [52]. Therefore, anticipatory anhedonia may be more closely linked to domains most salient in individuals' minds. We observed that *anticipatory anhedonia* was related to *low mood* and *suicidal ideation* among depressive nodes. Constantly expecting less pleasure may link to negative beliefs about the stability and significance of negative events [53, 54], which may connect to feelings of sadness and a sense of hopelessness about the future that results in suicidal ideation. This is consistent with qualitative research showcasing how anhedonia relates to depressed mood and reduced motivation to engage in activities and continue living [55, 56]. The association between anhedonia and suicidal ideation is particularly robust, with meta-analytic evidence pointing to anhedonia as a core risk factor for suicidal ideation [57]. With social anxiety nodes, we found that *anticipatory anhedonia* was linked to greater *avoidance of being the centre of attention* and less *fear of embarrassment*. Persistently expecting less pleasure, particularly in social contexts, may relate to worries about the negativity of future social events, as corroborated by qualitative reports of young people with social anxiety [58]. These worries could then lead to avoidance of situations wherein the individual is at risk of social evaluation [59, 60]. Conversely, such avoidance can also prevent individuals from learning that social situations can be enjoyable [59], which may contribute to anticipatory anhedonia. The association with less fear of embarrassment was an unexpected finding, as it appears inconsistent with models of social anxiety highlighting how negative expectations of future situations maintain social fears [59, 60]. Replications are needed to check the validity of this association.

In contrast to reward anticipation, reward consumption engages top-down and bottom-up processes, wherein behavioural and neural responses are a product of sensory-affective experiences and prior expectations [61, 62]. The involvement of momentary sensory-affective experiences may explain why consummatory anhedonia exhibited associations with not only the most salient symptom domains but also additional ones. *Consummatory anhedonia* was associated with *worthlessness/guilt* and *suicidal ideation* in our study. This may be because experiencing less pleasure daily may feed into negative beliefs about the self and the world, relating to worthlessness and suicidal ideation, respectively. Conversely, these beliefs may contribute to experiencing less pleasure generally. Supporting this, lived experience narratives have emphasised how a ‘stagnation of the present' corresponds with worthlessness, representing a general loss of enjoyment from life [56]. We also noted that *consummatory anhedonia* was associated with *concentration problems* and *sleep problems*, possibly because reduced enjoyment may contribute to difficulties engaging with activities, as well as struggling to unwind before bedtime. Supporting this, qualitative reports have indicated that individuals with anhedonia struggled to partake in effortful activities [55]. Disruptions in sleep and concentration may also impair one's ability to experience pleasure from daily activities, emphasising the potential utility of improving these problems to alleviate consummatory anhedonia [63]. Anhedonia's association with sleep difficulties is another robust finding in the literature [64, 65], and this has been shown to further contribute to suicidal ideation [66]. *Consummatory anhedonia* was associated with all social anxiety nodes, possibly due to the interplay between reduced pleasure and social contexts [67]. This may reflect how fears of embarrassment and avoidance of potential scrutiny may be connected to difficulties enjoying social activities [58].

Our findings suggest that anhedonia may be well conceptualised as a transdiagnostic symptom. Early models of depression and anxiety defined anhedonia as a uniquely depression-related symptom [68]. However, subsequent evidence suggested that social anxiety may be the exception to this rule, with socially anxious individuals demonstrating levels of anhedonia comparable to depression [12, 13, 69]. Concordant with this, previous network analyses have demonstrated that anhedonia shows strong associations across depression and social anxiety symptoms [29, 30]. Our study further supports this transdiagnostic perspective, delineating how anhedonia is intertwined with symptoms of depression and social anxiety.

Beyond anhedonia, our network analysis also identified other key associations. Cognitive-affective symptoms of depression—including *low mood, worthlessness/guilt* and *suicidal ideation*—were all strongly connected. This interconnectedness may imply the presence of potential depressive cycles; for example, feeling inadequate may lead to a recession in mood, which may then exacerbate worthlessness. This link between worthlessness and depressed mood has been demonstrated in longitudinal dynamic network studies [70]. Consistent with previous network analyses, *worthlessness/guilt* was a central node within the depressive community [71] and emerged as a strong node linking depression and social anxiety [72]. Depression and social anxiety may co-occur due to shared vulnerability arising from feelings of inadequacy, wherein negative beliefs about one's position in social hierarchies result in either submissive (i.e., socially anxious) or defeat (i.e., depressive) responses to negative events [73]. Another bridge symptom, *avoiding being the centre of attention*, has previously been linked to both depression [74] and social anxiety [59]. Supporting this, behavioural avoidance in social anxiety has been shown to predict the onset of depression [75], potentially due to links between avoidance and loneliness [76]. Given the high co-occurrence rates of depression and social anxiety [1], numerous symptoms likely connect the conditions, and this study provides insight into several symptoms that may be important to consider when understanding their co-occurrence.

Limitations of this study must be considered when interpreting its findings. The analysis was cross-sectional, precluding us from understanding the direction of associations and drawing causal inferences. Follow-up studies using longitudinal data are crucial if we are to identify precisely how activation spreads across these symptoms, to confirm the validity of targeting anhedonia in interventions. Furthermore, this study utilised a non-clinical sample, and the findings may not be generalisable to clinical populations. Nevertheless, a large proportion of our sample scored above the clinical cutoff for depression and social anxiety. The prevalence of these in the current sample was greater than the prevalence rates of depression [77, 78] and social anxiety [78] in young people in Norway, as well as prevalence rates of depression [79] and social anxiety [80] in young people worldwide. Given that these conditions are often viewed as dimensional [81, 82], based on the network theory of mental disorders [23], we may expect to see denser network structures and stronger edges between anhedonia and symptoms of depression and social anxiety in clinical samples. Future network analyses using clinical data are necessary to assess generalisability to individuals with more severe depression and social anxiety, and to evaluate the potential treatment relevance of targeting anhedonia. Our sample was also predominantly female, limiting the generalisability of our findings to individuals of other genders, particularly given potential gender differences in the manifestation of anhedonia [83, 84]. Future research should ensure that individuals across all genders are adequately represented in the data.

There are also certain limitations with respect to measurement. The measures used vary in their recall window, with Mini-SPIN and TEPS items referring to the past week, while PHQ-9 items refer to the past 2 weeks. While the impact of such measurement differences is not fully understood, these differences may have attenuated effects, wherein recency biases may have resulted in overestimated social anxiety and anhedonia levels (as well as associations) compared to depression; alternatively, social anxiety and anhedonia may be recalled more accurately given the recency, while depressive symptom estimates may be remembered with lower accuracy. Future studies may attempt to replicate our findings using measures with matched recall windows. Further, while the Mini-SPIN captures the core diagnostic features of social anxiety, it does not measure important social anxiety-related constructs, such as maintenance mechanisms. The field would benefit from examinations of how anhedonia links to a wider range of social anxiety symptoms, including self-focused attention and safety behaviours [59]. Studies should also explore additional dimensions of anhedonia, such as anhedonia relating to remembered pleasure and the distinction between social and physical anhedonia [17]. Using item-level indicators for anhedonia would offer the additional advantage of increasing granularity in our understanding with respect to specific aspects of different dimensions of anhedonia. Follow-up studies could also incorporate measures of additional related constructs (e.g., motivational difficulties) to disentangle these from anhedonia. Such examinations may offer greater precision in our understanding of anhedonia in depression and social anxiety.

Nevertheless, this study holds important strengths and implications. No known studies have examined differences in anticipatory and consummatory anhedonia in depression and social anxiety at the symptom level. Neuroimaging [19, 20] and behavioural [21, 22] studies have suggested that depression entails anticipatory, but not consummatory, anhedonia; instead, our study demonstrates that the nature of impairments may be more nuanced. This discrepancy may reflect differences in the level of analysis: while neuroimaging and experimental paradigms capture task-based indicators of hedonic response, the self-report measures used in this study likely capture the subjective experience of anhedonia, and it may be that consummatory anhedonia is pervasive on this subjective level. This underscores the importance of triangulating different levels of analysis. Our results present a critical advance in our understanding of the psychopathological expression of anhedonia in depression and social anxiety, particularly in light of the ongoing paradigm shift towards targeting anhedonia in interventions [11]. Albeit pending future longitudinal support, the transdiagnostic links of anticipatory and consummatory anhedonia found in the present study suggest that it may be an important symptom to consider when designing early interventions for depression and social anxiety, and there may be value in including strategies addressing both anticipatory anhedonia (e.g., pleasant event scheduling [31, 32]) and consummatory anhedonia (e.g., imaginal recounting [33, 34]) in interventions. Our findings also raise the potential for personalising early interventions depending on individuals' symptom risk profiles; for example, for a student indicating risk of low mood, it may be of value to account for anticipatory anhedonia. Such approaches may serve to reduce the risk of emerging symptoms becoming clinically significant and impairing. It is worth exploring these potential avenues further, as these can be especially beneficial for improving the mental health for university students, who are particularly at risk for experiencing such difficulties [7]. Applying a network analytic approach to a large sample of students allowed for the examination of interrelationships between these constructs in a methodical and data-driven manner. This method allowed us to gain insight into the most pervasive symptoms of depression and social anxiety in university students, highlighting potential symptoms to consider when crafting interventional approaches for this vulnerable population [7, 8].

## 5. Conclusions

Overall, these findings contribute to a more granular understanding social anxiety and depressive symptomatology in university students. They suggest that anhedonia may be conceptualised as a transdiagnostic symptom relevant to depressive and social anxiety symptoms in young people, and if replicated in longitudinal studies, anhedonia may serve as an important symptom to account for when designing early interventions for depression and social anxiety. The results further exemplify the importance of differentiating between dimensions of anhedonia, suggesting that clinical assessments and interventions may need to incorporate strategies addressing both anticipatory and consummatory anhedonia. This distinction may enhance the precision of early identification and intervention strategies for depression and social anxiety, serving as a meaningful step towards improving mental health outcomes for university students.

## Figures and Tables

**Table 1:** Sample descriptive statistics for included measures.

Measure	Range	*M*	*SD*	*N* above clinical cutoff
Patient Health Questionnaire (PHQ-9)	0–27	8.16	5.82	194 (28.87%)
Mini Social Phobia Inventory (Mini-SPIN)	0–12	3.52	3.15	127 (18.90%)
Temporal Experience of Pleasure Scale (TEPS) – anticipatory	10–60	29.5	9.32	N/A
Temporal Experience of Pleasure Scale (TEPS) – consummatory	8–46	21.31	7.95	N/A

*Note*: The table provides the sample range, mean (*M*), standard deviation (*SD*), and number of participants (*N*) scoring above the clinical cutoff for depression (cutoff = 10) and social anxiety (cutoff = 6) on the included measures. The PHQ-9 measured depression symptoms (range 0–27). The Mini-SPIN measured social anxiety symptoms (range 0–12). The TEPS measured anticipatory anhedonia (range 10–60) and consummatory anhedonia (range 8–48).

## Data Availability

Access to the data can be granted from the principal investigator Omid V. Ebrahimi (omid.ebrahimi@psy.ox.ac.uk) following approval of a suggested project plan for the use of data granted by The Regional Committee for Medical and Health Research Ethics (REK).

## Endnotes

^1^We post hoc restricted age to ≤24, to align with literature about the age characterisation of young people [36]. This was not pre-registered, and the decision was made after examining the data because the sample was older than anticipated and did not align with our rationale of informing potential early interventional approaches for young people experiencing depressive and social anxiety symptoms. As a sensitivity analysis, we compared the edge weight matrices of the full unfiltered sample with the filtered sample. The edge weights matrix for the unfiltered sample is available in the Supporting Information (Table S3). The correlation between edge weight matrices of the filtered (*n* = 672) and unfiltered sample (*n* = 1256) networks was high, *r* = .98, suggesting that our model was sufficiently powered and robust to changes in sample size.

^2^In our pre-registration, we stated that we would use the *GGMModSelect* model selection algorithm [49]. However, filtering the sample based on age reduced the sample size from 1256 to 672. For smaller samples, published guidelines based on simulations [45] recommend using *EBICglasso*. These simulations indicated that *EBICglasso* is sufficiently able to detect the true network structure with sample sizes of *n* = 600.

## Supporting Information

Supporting Information Table S1 presents descriptive statistics for each node in the network, including node-wise range, mean, and standard deviation for depression, social anxiety, and anhedonia. Table S2 presents edge weights/partial correlation coefficients for each pair of nodes in the main network presented in the manuscript, for students ≤ 24 years. Table S3 presents edge weights/partial correlation coefficients for each pair of nodes for the sensitivity analysis with the full sample, without filtering for age. Figure S4 depicts the network visualisation for associations with anticipatory anhedonia, hiding associations with consummatory anhedonia. Figure S5 depicts the network visualisation for associations with consummatory anhedonia, hiding associations with anticipatory anhedonia. Figure S6 illustrates the plot for the centrality stability estimates for expected influence and bridge expected influence metrics, depicting the correlation between the values of bootstrapped cases and the original sample. Figure S7 illustrates the plot depicting the bootstrapped confidence intervals to estimate edge weight accuracy. Figure S8 illustrates the plot showing the results of the bootstrapped difference tests to estimate edge weight accuracy.
