# Supplementary material for: Differential Associations of Anticipatory and Consummatory Anhedonia With Depression and Social Anxiety Symptoms: A Network Analysis of University Students
Source: Depress Anxiety. 2025 Oct 30;2025:5674096. doi: 10.1155/da/5674096 (PMC12591812; doi:10.1155/da/5674096)
Supplement: Supporting Information — Table S1 presents descriptive statistics for each node in the network, including node-wise range, mean, and standard deviation for depression, social anxiety, and anhedonia. Table S2 presents edge weights/partial correlation coefficients for each pair of nodes in the main network presented in the manuscript, for students ≤ 24 years. Table S3 presents edge weights/partial correlation coefficients for each pair of nodes for the sensitivity analysis with the full sample, without filtering for age. Figure S4 depicts the network visualisation for associations with anticipatory anhedonia, hiding associations with consummatory anhedonia. Figure S5 depicts the network visualisation for associations with consummatory anhedonia, hiding associations with anticipatory anhedonia. Figure S6 illustrates the plot for the centrality stability estimates for expected influence and bridge expected influence metrics, depicting the correlation between the values of bootstrapped cases and the original sample. Figure S7 illustrates the plot depicting the bootstrapped confidence intervals to estimate edge weight accuracy. Figure S8 illustrates the plot showing the results of the bootstrapped difference tests to estimate edge weight accuracy. [file 5674096.f1.docx]

**Supporting Information**

**S1.** Descriptive statistics for each node in the network

| Measure | Item | Node label | Range | *M* | *SD* |
| --- | --- | --- | --- | --- | --- |
| Patient Health Questionnaire (PHQ-9) | Feeling down, depressed, or hopeless | Low mood | 0-3 | 0.92 | 0.86 |
|  | Trouble falling or staying asleep, or sleeping too much | Sleep problems | 0-3 | 1.22 | 0.99 |
|  | Feeling tired or having little energy | Low energy | 0-3 | 1.37 | 0.90 |
|  | Poor appetite or overeating | Appetite changes | 0-3 | 1.04 | 1.01 |
|  | Feeling bad about yourself or that you are a failure or have let yourself or your family down | Worthlessness/guilt | 0-3 | 0.97 | 0.96 |
|  | Trouble concentrating on things, such as reading the newspaper or watching television | Concentration problems | 0-3 | 1.05 | 0.97 |
|  | Moving or speaking so slowly that other people could have noticed. Or the opposite, being so fidgety or restless that you have been moving around a lot more than usual | Psychomotor agitation/slowing | 0-3 | 0.30 | 0.63 |
|  | Thoughts that you would be better off dead, or of hurting yourself | Suicidal ideation | 0-3 | 0.30 | 0.67 |
| Mini Social Phobia Inventory (Mini-SPIN) | Fear of embarrassment causes me to avoid doing things or speaking to people. | Avoiding embarrassment | 0-4 | 1.15 | 1.10 |
|  | I avoid activities in which I am the center of attention. | Avoiding being centre of attention | 0-4 | 1.00 | 1.18 |
|  | Being embarrassed or looking stupid are among my worst fears. | Fear of Embarrassment | 0-4 | 1.37 | 1.30 |
| Temporal Experience of Pleasure Scale (TEPS) | Aggregate of anticipatory pleasure items | Anticipatory anhedonia | 10-60 | 29.5 | 9.32 |
|  | Aggregate of consummatory pleasure items | Consummatory anhedonia | 8-46 | 21.31 | 7.95 |

*Note*. The table provides the range, mean (*M*), and standard deviation (*SD*) for each node in the network. Items from the PHQ-9 measured depression symptoms (range 0-3). Items from the Mini-SPIN measured social anxiety symptoms (range 0-4). Items from the TEPS measured anticipatory anhedonia (range 10-60) and consummatory anhedonia (range 8-48)

**S2.** Edge weights matrix for the filtered sample: main analysis

| Node | | 1 | 2 | 3 | 4 | 5 | 6 | 7 | 8 | 9 | 10 | 11 | 12 | 13 |
| --- | --- | --- | --- | --- | --- | --- | --- | --- | --- | --- | --- | --- | --- | --- |
| 1 | Mood | — |  |  |  |  |  |  |  |  |  |  |  |  |
| 2 | Sleep | 0.090 | — |  |  |  |  |  |  |  |  |  |  |  |
| 3 | Energy | 0.222 | 0.295 | — |  |  |  |  |  |  |  |  |  |  |
| 4 | Appetite | 0.072 | 0.152 | 0.142 | — |  |  |  |  |  |  |  |  |  |
| 5 | Worth | 0.276 | 0.030 | 0.083 | 0.093 | — |  |  |  |  |  |  |  |  |
| 6 | Conc | 0.006 | 0.120 | 0.163 | 0.162 | 0.104 | — |  |  |  |  |  |  |  |
| 7 | Motor | 0.078 | 0.092 | 0.023 | 0.031 | 0.071 | 0.159 | — |  |  |  |  |  |  |
| 8 | Suicidal | 0.204 | 0.075 | 0.000 | 0.070 | 0.201 | 0.000 | 0.038 | — |  |  |  |  |  |
| 9 | AvoidEmb | 0.033 | 0.000 | 0.000 | 0.000 | 0.101 | 0.014 | 0.000 | 0.000 | — |  |  |  |  |
| 10 | AvoidAtt | 0.014 | 0.000 | 0.003 | 0.067 | 0.031 | 0.079 | 0.022 | 0.047 | 0.424 | — |  |  |  |
| 11 | FearOfEmb | 0.018 | 0.008 | 0.000 | 0.014 | 0.033 | 0.035 | -0.017 | 0.000 | 0.407 | 0.198 | — |  |  |
| 12 | AntiAnh | 0.099 | 0.000 | 0.000 | 0.000 | 0.000 | 0.000 | 0.000 | 0.124 | 0.000 | 0.025 | -0.056 | — |  |
| 13 | ConsAnh | 0.001 | 0.042 | 0.000 | 0.000 | 0.023 | 0.045 | 0.000 | 0.011 | 0.040 | 0.008 | 0.058 | 0.595 | — |

*Note:* This table provides the partial correlation coefficients between all pairs of nodes in the network presented in the manuscript, for students aged ≤ 24 years.

**S3.** Edge weights matrix for the full sample: sensitivity analysis

| Node | | 1 | 2 | 3 | 4 | 5 | 6 | 7 | 8 | 9 | 10 | 11 | 12 | 13 |
| --- | --- | --- | --- | --- | --- | --- | --- | --- | --- | --- | --- | --- | --- | --- |
| 1 | Mood | — |  |  |  |  |  |  |  |  |  |  |  |  |
| 2 | Sleep | 0.075 | — |  |  |  |  |  |  |  |  |  |  |  |
| 3 | Energy | 0.189 | 0.284 | — |  |  |  |  |  |  |  |  |  |  |
| 4 | Appetite | 0.091 | 0.149 | 0.119 | — |  |  |  |  |  |  |  |  |  |
| 5 | Worth | 0.301 | 0.017 | 0.106 | 0.091 | — |  |  |  |  |  |  |  |  |
| 6 | Conc | 0.080 | 0.119 | 0.159 | 0.143 | 0.138 | — |  |  |  |  |  |  |  |
| 7 | Motor | 0.057 | 0.066 | 0.046 | 0.059 | 0.050 | 0.152 | — |  |  |  |  |  |  |
| 8 | Suicidal | 0.193 | 0.047 | 0.000 | 0.078 | 0.165 | 0.000 | 0.086 | — |  |  |  |  |  |
| 9 | AvoidEmb | 0.013 | 0.009 | 0.000 | 0.000 | 0.094 | 0.048 | 0.000 | 0.000 | — |  |  |  |  |
| 10 | AvoidAtt | 0.051 | 0.000 | 0.000 | 0.057 | 0.010 | 0.041 | 0.048 | 0.034 | 0.410 | — |  |  |  |
| 11 | FearOfEmb | 0.017 | 0.000 | 0.000 | 0.002 | 0.022 | 0.011 | 0.000 | 0.000 | 0.419 | 0.204 | — |  |  |
| 12 | AntiAnh | 0.065 | 0.001 | 0.010 | 0.000 | 0.000 | 0.000 | 0.000 | 0.128 | 0.000 | 0.018 | -0.017 | — |  |
| 13 | ConsAnh | 0.023 | 0.054 | 0.010 | 0.000 | 0.041 | 0.016 | 0.000 | 0.015 | 0.041 | 0.028 | 0.022 | 0.592 | — |

*Note:* This table provides the partial correlation coefficients between all pairs of nodes for the full sample, without filtering for age. The correlation between edge weight matrices of the filtered and full samples was high, *r* = .98.

**S4.** Network visualisation for associations with anticipatory anhedonia

**
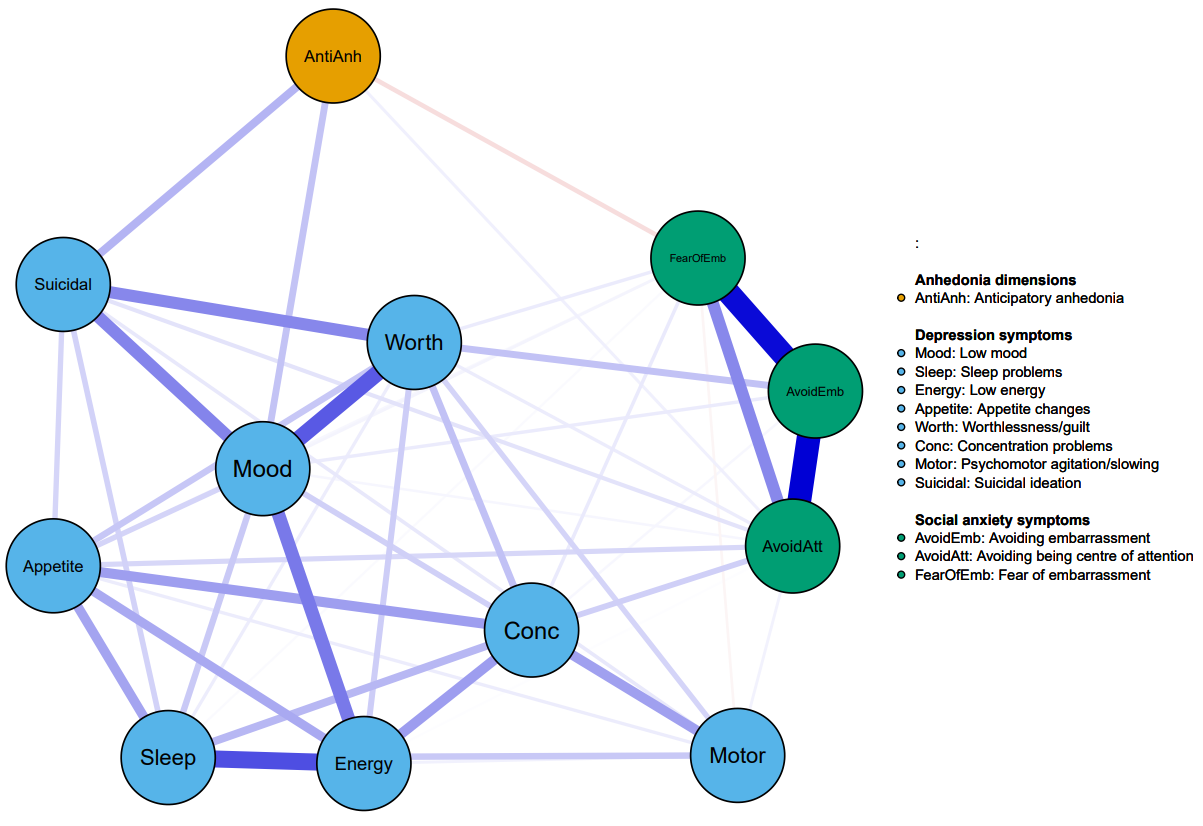
**

**S5.** Network visualisation for associations with consummatory anhedonia

**
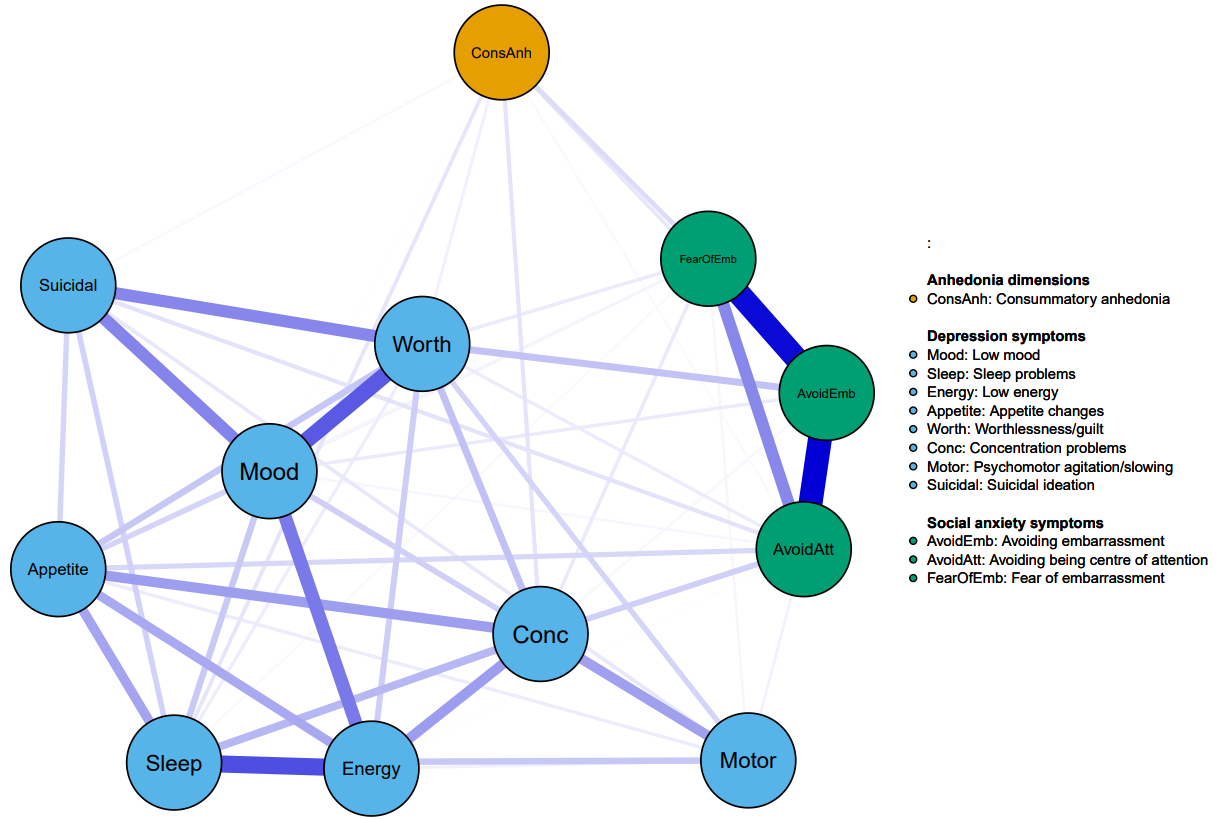
**

**S6.** Centrality stability estimate


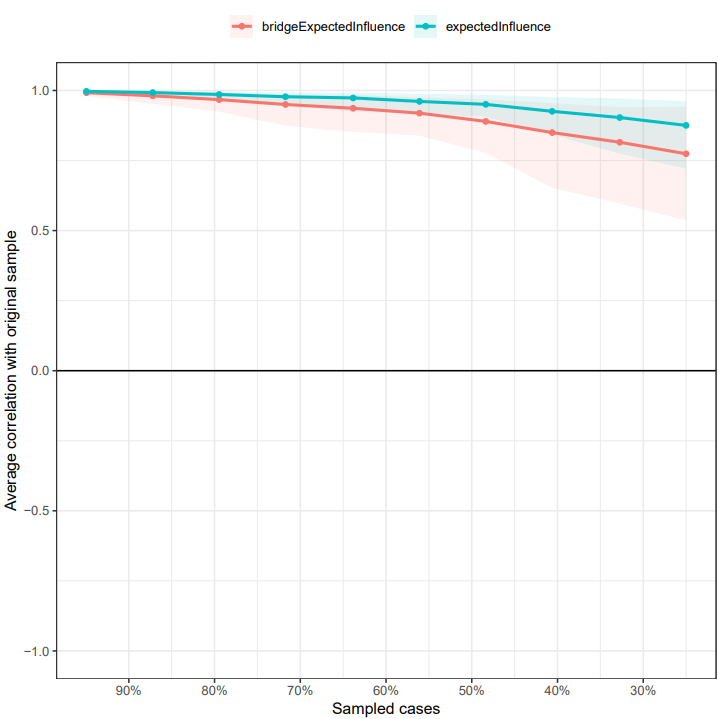


**S7.** Bootstrapped confidence intervals for edge weight accuracy

**
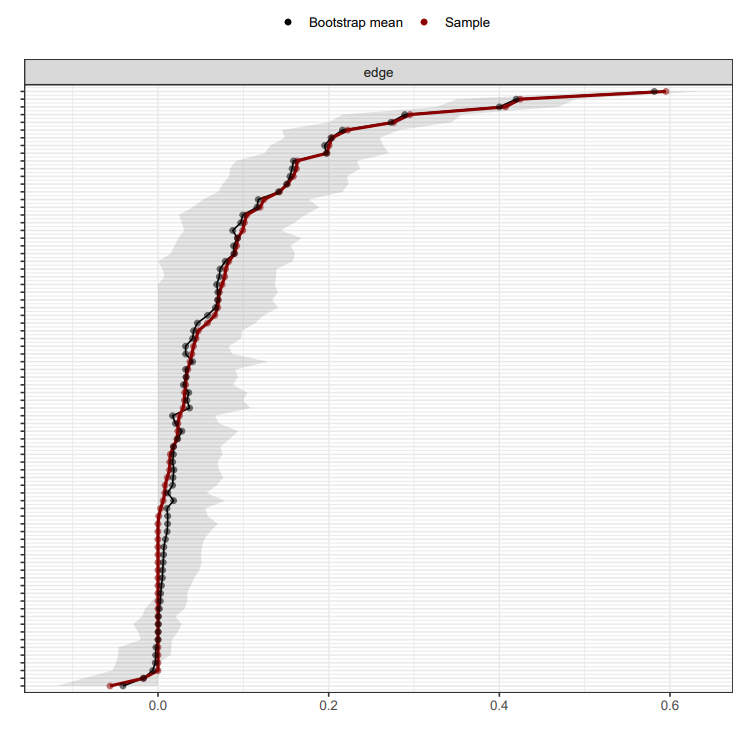
**

**S8.** Bootstrapped difference tests for edge weight accuracy

**
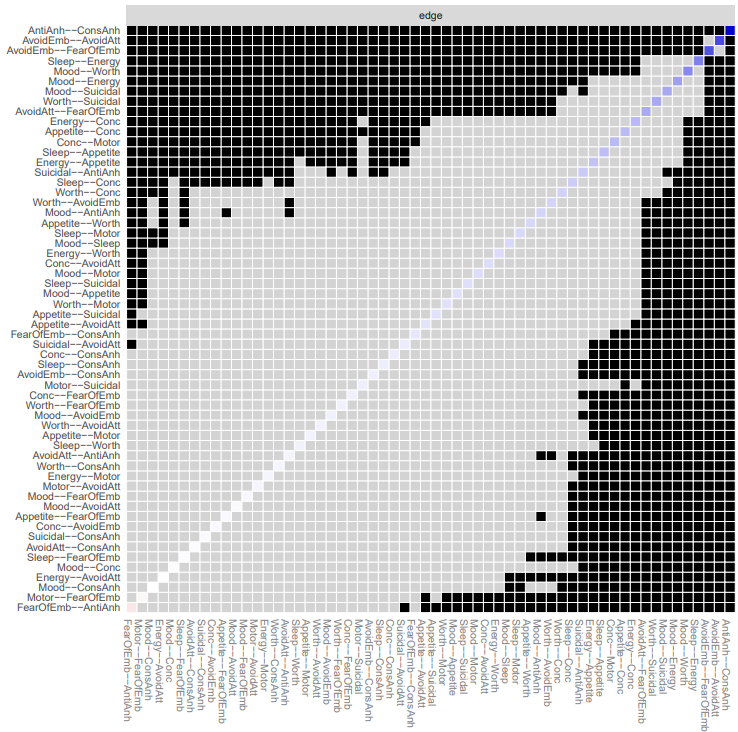
**
